# Supplementary material for: Deletion of wfs1 Impairs Oligodendrocyte Precursor Cells Dorsal Distribution and Myelination Through the wfs1-hmgcs1 Axis in Zebrafish
Source: Biology (Basel). 2026 Aug 16;15(16):1402. doi: 10.3390/biology15161402 (PMC13510151; doi:10.3390/biology15161402)
Supplement: Supplementary file 1 [file biology-15-01402-s001.zip › File S1-Transcriptome sequencing data and differentially expressed genes (DEGs) in wfs1-- zebrafish at 5 dpf/2.KEGG/wfs1KOvsWT/DOWN/src/dre00520.html]

dre00520


- dre:406339

- Down regulated genes

406339\_1(-1.38680349052238)

- dre:406339

- Down regulated genes

406339\_1(-1.38680349052238)

- dre:563138

- Down regulated genes

563138(-1.61689006980077)

- dre:100007686

- Down regulated genes

100007686(-1.06018652619591)

- dre:406339

- Down regulated genes

406339\_1(-1.38680349052238)

- dre:406339

- Down regulated genes

406339\_1(-1.38680349052238)

- dre:406339

- Down regulated genes

406339\_1(-1.38680349052238)

- dre:406339

- Down regulated genes

406339\_1(-1.38680349052238)

Close
